# A paracrine interaction between granulosa cells and leukocytes in the preovulatory follicle causes the increase in follicular G-CSF levels

**DOI:** 10.1007/s10815-020-01692-y

**Published:** 2020-01-18

**Authors:** Laure Noël, Maïté Fransolet, Nathalie Jacobs, Jean-Michel Foidart, Michelle Nisolle, Carine Munaut

**Affiliations:** 1 Centre de Procréation Médicalement Assistée, University of Liege, site CHR Liege, Boulevard du 12ème de Ligne 1, 4000 Liege, Belgium; 2 Laboratory of Tumor and Development Biology, University of Liege, 4000 Liege, Belgium; 3 Laboratory of Cellular and Molecular Immunology, GIGA Research, University of Liege, 4000 Liege, Belgium

**Keywords:** G-CSF, Ovary, Granulosa cells, Ovulation, Leukocytes

## Abstract

**Objective:**

Follicular granulocyte colony-stimulating factor (G-CSF) is a new biomarker of oocyte quality and embryo implantation in in vitro fertilization (IVF) cycles. Its role in reproduction is poorly understood. Our study aimed to investigate the mechanisms and cells responsible for G-CSF production in the preovulatory follicle.

**Design:**

Laboratory research study.

**Setting:**

Single-center study.

**Interventions:**

Granulosa cells and leukocytes were isolated from the follicular fluids (FF) or the blood of women undergoing IVF and from the blood of a control group of women with spontaneous ovulatory cycles to perform cocultures.

**Main outcome measure:**

G-CSF-secreted protein was quantified in the conditioned media of cocultures.

**Results:**

G-CSF secretion was considerably increased in cocultures of granulosa cells and leukocytes. This effect was maximal when leukocytes were isolated from the blood of women in the late follicular phase of the menstrual cycle or from the FF of women undergoing IVF. The leukocyte population isolated from the FF samples of women undergoing IVF had a higher proportion of granulocytes than that isolated from the corresponding blood samples. Leukocytes induced the synthesis and secretion of G-CSF by granulosa cells. Among a range of other FF cytokines/chemokines, only growth-regulated oncogene alpha (GROα) was also increased.

**Conclusion:**

The notable rise in G-CSF at the time of ovulation coincides with the accumulation of follicular granulocytes, which stimulate G-CSF production by granulosa cells via paracrine interactions. High follicular G-CSF concentrations may occur in follicles with optimal granulosa–leukocyte interactions, which could explain the increased implantation rate of embryos arising from these follicles.

**Electronic supplementary material:**

The online version of this article (10.1007/s10815-020-01692-y) contains supplementary material, which is available to authorized users.

## Introduction

Follicular fluid (FF) surrounds the oocyte and constitutes the milieu in which signaling molecules circulate between granulosa cells and the oocyte during folliculogenesis and oocyte maturation [1]. FFs are collected with the oocytes during in vitro fertilization (IVF) procedures and are discarded after oocyte retrieval. Therefore, FF represents a source of non-invasive biomarkers of oocyte quality. A competent oocyte is able to be fertilized and to support the first steps of embryo development [2]; however, only 30% of retrieved oocytes will develop into a good quality embryo and 5% will result in a live birth [3, 4]. Oocyte quality assessment remains a challenge in IVF, since morphology is poorly predictive of oocyte competence [5].

Ovulation has been compared to a controlled inflammatory reaction with local hyperemia, connective tissue degradation, chemokine/cytokine accumulation, and leukocyte chemotaxis in the preovulatory follicle [6–9]. G-CSF is a multifunctional cytokine, best known for stimulating the production and differentiation of neutrophils during hematopoiesis and promoting their activation, with key roles in immunity and inflammatory responses [10–12]. G-CSF also has a recognized role in several aspects of human reproduction [13–16]. In a recent trial with an ultra-sensitive ELISA specifically designed for the quantification of follicular G-CSF, the probability of successful implantation was 3.3-fold higher for embryos derived from follicles with high G-CSF levels than for those derived from follicles with low G-CSF levels [17].

However, the mechanisms and role of follicular G-CSF in oocyte maturation and ovulation remains poorly understood, including the understanding of fundamental aspects regarding the local production and secretion of G-CSF. Serum G-CSF level peaks at the time of spontaneous ovulation [18] or ovulation induction during ovarian stimulation with gonadotrophins [19, 20]. The intrafollicular G-CSF production by granulosa cells and its higher concentration in FF than in serum [21] suggest that G-CSF might play a paracrine role within the follicular environment. It has been demonstrated that human leukocytes, and in particular neutrophils, accumulate in the follicular wall just before ovulation [9, 22]. These data suggest that G-CSF, with its ability to recruit and activate neutrophils, might play a role in the mechanism of oocyte maturation and subsequent ovulation.

We were therefore interested in further evaluating the paracrine interaction between granulosa cells and leukocytes to enhance preovulatory G-CSF synthesis and secretion.

## Materials and methods

### Study design

**Fig. 1:**
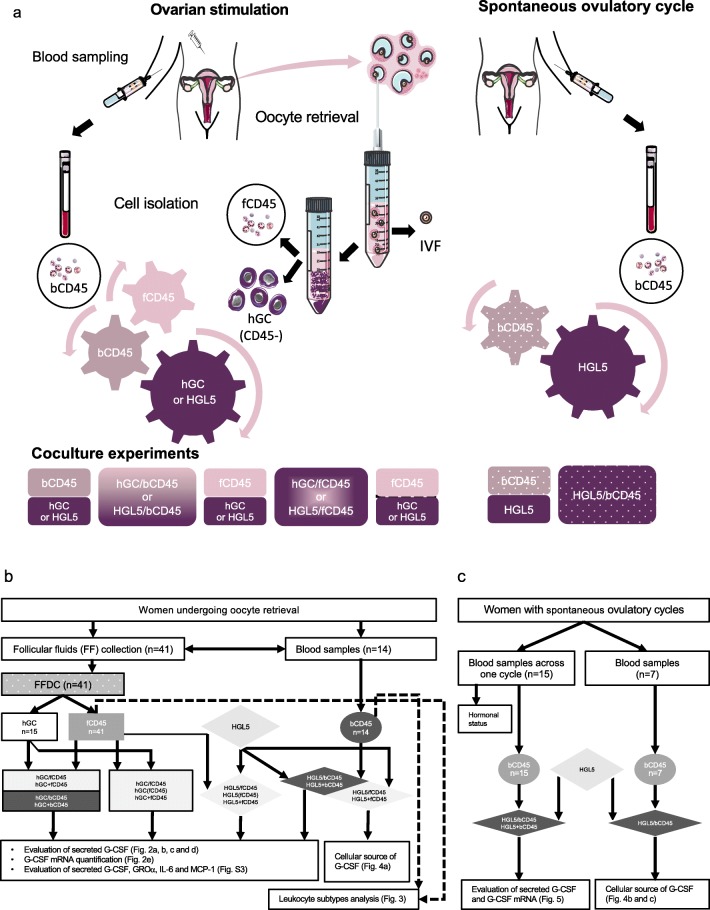
Study design. CD45-negative human granulosa cells (hGC) and follicular CD45-positive (fCD45) leukocytes were isolated from the FF of women undergoing ovarian stimulation for IVF, and blood CD45-positive (bCD45) leukocytes were isolated from their peripheral blood at the time of oocyte retrieval (**a** and **b**). bCD45 leukocytes were also isolated from the peripheral blood of women with spontaneous ovulatory cycles (**a** and **c**). HGL5 cells correspond to a human granulosa-derived cell line. Several culture experiments were performed: separate cultures (hGC, HGL5, fCD45, or bCD45); cocultures with cell contact (hGC/CD45 or HGL5/CD45) and cocultures without cell contact (hGC(CD45) or HGL5(CD45)), which were evaluated for secreted G-CSF by ELISA assay (**b** and **c**). G-CSF mRNA quantification was further performed on cell culture assays using the HGL5 cell line (**b** and **c**). hGC + CD45 and HGL5 + CD45 represent the algebraic sum of G-CSF secreted by separately cultured hGC and CD45 cells or by separately cultured HGL5 and CD45 cells (**b** and **c**)

This was a single-center laboratory research study (Centre de Procréation Médicalement Assistée, CHR Liege Hospital, Liege University). It was approved by the hospital Ethics committee (CE412/1508). We used an in vitro system to test the mechanisms of follicular G-CSF production by granulosa cells and their paracrine interaction with leukocytes. CD45-negative human granulosa cells (hGC) and CD45-positive follicular (fCD45) leukocytes were isolated from the FF of women undergoing IVF, and CD45-positive blood (bCD45) leukocytes were isolated from their peripheral blood (Fig. 1a and b). Blood leukocytes (bCD45) were also isolated from the peripheral blood of women with spontaneous ovulatory cycles (Fig. 1a and c). A human granulosa-derived cell line (HGL5) was also used as a model for primary hGC. The HGL5 cell line maintains many of the functions of primary hGC [23], including G-CSF production.

Secreted levels of G-CSF (and a variety of other cytokines and chemokines) in the conditioned media were analyzed by ELISA assays following separate cultures (hGC, HGL5, fCD45, or bCD45 cells); cocultures with cell contact (hGC/CD45 or HGL5/CD45); and cocultures without cell contact (hGC(CD45) or HGL5(CD45)). We also assessed the leukocyte subtypes in the populations of fCD45 and bCD45 cells isolated from women undergoing IVF and the cellular source of G-CSF production in cocultures of HGL5 cells and leukocytes. Finally, cocultures of HGL5 with bCD45 cells were performed to evaluate the influence of the menstrual cycle phase on G-CSF secretion. G-CSF mRNA quantification was further performed on cell culture assays using the HGL5 cell line.

### Patients

Samples of FF were obtained from 41 women aged 23–39 years who underwent IVF/ICSI for primary infertility due to male factor or polycystic ovary syndrome (PCOS) or unexplained infertility following hormonal blood tests, ultrasound examination and sperm analysis (Fig. 1b). All patients underwent an antagonist protocol for ovarian stimulation. FF was collected from each of the women to perform 41 independent cell isolations. Peripheral blood samples were also collected from 14 of these women at the time of oocyte pick up (Fig. 1b). In addition, 15 blood samples from 3 women with spontaneous ovulatory and regular menstrual cycles (27 to 35 days) were collected during the follicular, late follicular, ovulatory, or luteal phases of one menstrual cycle, based on the serum levels of estradiol, luteinizing hormone, and progesterone (Fig. 1c and Table S1). Seven additional blood samples were obtained from these women with spontaneous ovulatory cycles, at various stages of their menstrual cycles. Written informed consent was obtained from all women included in the study.

### Granulosa cell and leukocyte isolation from follicular fluids

The protocol of human granulosa cells (hGC) and leukocyte isolation from FF was adapted from Shi et al. [24], as described previously by our team [25]. Briefly, FF was centrifuged after oocyte recovery. After the supernatant was removed, the pellet was resuspended in an enzymatic solution to digest clusters of cells. FF-derived cells (FFDC) were obtained by density gradient centrifugation over Ficoll-Paque Plus (GE Healthcare, Little Chalfont, Buckinghamshire, England). Viable cells were counted using the trypan blue exclusion method.

Magnetic-activated cell sorting (MACS) was performed to obtain the following two cell populations: CD45-negative cells representing human granulosa cells (hGC) and CD45-positive cells representing follicular leukocytes (fCD45). Flow cytometry with a FACSCanto II device (BD Biosciences) was performed to analyze cell populations. The median proportions of fCD45 cells and CD45-negative hGC in the FFDC population were 31% (P25 = 18; P75 = 53) and 69% (P25 = 47; P75 = 82), respectively. hGC cells were contaminated with fCD45 cells at a proportion of 2% (median proportion of hGC cells = 98.0% (P25 = 97.8; P75 = 99.0)), and fCD45 cells were contaminated with CD45-negative cells at a proportion of 14.5% (median proportion of fCD45 cells = 85.5% (P25 = 79; P75 = 89.2)). Cell viability was confirmed by flow cytometry using propidium iodide (Miltenyi Biotec).

### Peripheral blood mononuclear cell isolation

Peripheral blood samples were collected in EDTA tubes. Blood (5 ml) was layered on 5 ml of Ficoll-Paque Plus before centrifugation at 500*g* for 20 min. The superficial phase of the plasma was removed. The interphase containing peripheral blood mononuclear cell (PBMC) was collected and washed twice with phosphate-buffered saline (PBS). Viable cells were counted using trypan blue exclusion. Then, MACS of PBMC was performed, following the procedure described for FFDC [25]. A population of CD45-positive cells called blood leukocytes (bCD45) was obtained, following the protocol described previously.

### Cell culture reagents

FFDC, hGC, follicular and blood leukocytes (fCD45 and bCD45), and the human granulosa-derived cell line HGL5 [26] were maintained in Dulbecco’s-modified Eagle’s medium/F-12 with GlutaMAX, supplemented with 10% heat-inactivated fetal bovine serum and 1% penicillin–streptomycin (100 IU/ml penicillin and 100 μg/ml streptomycin). In addition, the medium for FFDC and hGC was supplemented with 1% ITS (6.25 μg/ml insulin, 6.25 μg/ml transferrin and 6.25 μg/ml selenium). Culture reagents were all purchased from Gibco (Thermo Fisher Scientific, Waltham, MA, USA). Cells were cultured in a traditional humidified incubator supplied with room air (20% oxygen and 75% nitrogen) buffered with 5% CO_2_ and set to 37 °C.

### Cell culture assays

To assess the interaction between granulosa cells (HGL5 cells or hGC) and leukocytes (fCD45 or bCD45 cells) in terms of G-CSF production, cells were either cultured separately or cocultured for 48 h. fCD45 and bCD45 cells were isolated from the FF or the peripheral blood of women undergoing IVF. For separate cultures, HGL5 cells or hGC or fCD45 or bCD45 cells were seeded in 12-well plates at a density of 5 × 10^5^ cells per well. For cocultures, 5 × 10^5^ HGL5 cells (or hGC) were seeded with 5 × 10^5^ fCD45 or bCD45 cells per well. Cocultures of HGL5 (or hGC) and fCD45 without cell contact were also performed by using plate inserts with a 0.4 mm pore size (ThinCert, Greiner Bio-One, Vilvoorde, Belgium); 5 × 10^5^ HGL5 (or hGC) cells were seeded into the bottom chamber and 5 × 10^5^ fCD45 cells were seeded into the upper chamber. In each experiment, hGC cells were cocultured with fCD45 or bCD45 cells isolated from the same woman. After 48 h, conditioned media were collected for the assessment of G-CSF secretion by ELISA assays and total RNA was extracted from the cultured cells to determine the mRNA levels.

To assess the source of G-CSF secretion, cocultures of HGL5 and fCD45 cells were performed with monensin. HGL5 and fCD45 cells were individually exposed to monensin (2 μM, Invitrogen, Thermo Fisher Scientific) overnight. Control HGL5 and control fCD45 cells, i.e., cells that were isolated from the FF of the same woman, were not exposed to monensin. Then, the media were removed, the cells were washed with fresh medium, and the following 4 cocultures of 5 × 10^5^ HGL5 and 5 × 10^5^ fCD45 cells were incubated for 8 h in 12-well plates: the untreated control coculture with no previous exposure to monensin; the coculture with both cell types pretreated with monensin; the coculture with monensin-pretreated HGL5 cells and untreated control fCD45 cells; and the coculture of untreated control HGL5 and monensin-pretreated fCD45. After 8 h, conditioned media were collected for the assessment of G-CSF secretion.

To further evaluate the source of G-CSF secretion, cocultures of HGL5 and bCD45 cells from women with spontaneous ovulatory cycles were performed for 24 h with different proportions of each cell type in 12-well plates: 2.5 × 10^5^ HGL5 cells and 7.5 × 10^5^ bCD45 cells; 5 × 10^5^ HGL5 cells and 5 × 10^5^ bCD45 cells; or 7.5 × 10^5^ HGL5 cells and 2.5 × 10^5^ bCD45 cells. After 24 h, conditioned media were collected for the assessment of G-CSF secretion and total RNA was extracted from the cultured cells.

To assess the effect of the menstrual cycle on G-CSF secretion, coculture experiments were performed with HGL5 and bCD45 cells isolated from the peripheral blood of women with spontaneous ovulatory cycles, at the follicular, late follicular, ovulatory, or luteal phases of one menstrual cycle. A total of 5 × 10^5^ HGL5 cells and 5 × 10^5^ bCD45 cells were either cultured separately or cocultured for 48 h in 12-well plates. After 48 h, conditioned media were collected for the assessment of G-CSF secretion and total RNA was extracted from the cultured cells.

Each cell culture assay was reproduced at least 3 times in independent experiments using cells isolated from separate women.

### Flow cytometry analysis of leukocyte subtypes

Single-cell suspensions of follicular and blood CD45-positive cells were obtained from FF or the peripheral blood of 5 women on the day of oocyte retrieval. Cell viability was assessed with the fixable viability stain 780 (FVS780). The anti-human antibodies used for surface staining of leukocytes are detailed in the supplemental data (Table S2). Cells were stained for 30 min at 4 °C. All antibodies and FVS780 were purchased from BD Biosciences (Franklin Lakes, NJ, USA). The BD LSRFORTESSA cell analyzer and the BD FACS Diva software version 8.0 (BD Biosciences) were used to perform flow cytometry. Flow cytometry standard files (FCS files) were further analyzed using FLOWJO version 10 software. The gating strategy used to determine the leukocyte subtypes is shown in the supplemental data (Fig. S1).

### ELISAs for the quantification of secreted G-CSF, GROα, MCP-1, and IL-6 and multianalyte ELISArray kits for the qualitative evaluation of secreted human cytokines and chemokines

Conditioned media from cultured cells were centrifuged at 15,000*g* for 5 min and supernatants were frozen at − 80 °C. The concentrations of G-CSF, growth-regulated oncogene alpha (GROα), monocyte chemoattractant protein-1 (MCP-1), and interleukin 6 (IL-6) were evaluated using specific ELISA assays for G-CSF, GROα, MCP-1 (Duo Set ELISA, R&D Systems, Minneapolis, MN, USA) and IL-6 (Human IL-6 CytoSet, Invitrogen), following the manufacturers’ protocols. The secreted levels of IL-2, IL-4, IL-5, IL-6, IL-10, IL-12, IL-13, IL-17A, IFNγ, TNFα, G-CSF, and TGF-β1 and of IL-8, MCP-1, RANTES, MIP-1α, MIP-1β, IP-10, I-TAC, MIG, Eotaxin, TARC, MDC, and GROα were qualitatively assessed with the human Th1/Th2/Th17 cytokines and the human common chemokines multianalyte ELISArray kits, respectively (Qiagen, Hilden, Germany). The quantification was performed in duplicate for each analyzed culture medium.

### Real-time PCR for mRNA quantification

Total RNA was extracted using the High Pure RNA Isolation Kit (Roche Life Science, Basel, Switzerland), following the manufacturer’s protocol. Then, 200 nanograms of RNA was reverse transcribed into cDNA using the Transcriptor First Strand cDNA Synthesis Kit (Roche Life Science). Real-time quantitative PCR was performed using specific primers and Brilliant SYBR GREEN QPCR master mix (Roche Life Science) for the 3 housekeeping genes (B2M, RPL13A, and TBP) or the probe 43 for hG-CSF from the Universal Probe Library (Roche Life Science) on a LightCycler® 480 (Roche Life Science). Sequence primers for the target genes were B2M forward 5′-TGCTGTCTCCATGTTTGATGTATCT-3′ and reverse 5′-TCTCTGCTCCCCACCTCTAAGT-3′; RPL13A forward 5′-CCTGGAGGAGAAGAGGAAAGAGA-3′ and reverse 5′-TTGAGGACCTCTGTGTATTTGTCAA-3′; TBP forward 5′-TGCACAGGAGCCAAGAGTGAA-3′ and reverse 5′-CACATCACAGCTCCCCACCA-3′; and hG-CSF forward 5′-CACCTACAAGCTGTGCCACC-3′ and reverse 5′-TTCCCAGTTCTTCCATCTGCT-3′. Gene expression values were normalized to the geometric mean of the three housekeeping genes, and mRNA expression levels were quantified using the ΔCT method [27].

### Statistical analysis

All quantitative variables followed a non-normal distribution and are expressed as the medians ± interquartile ranges (P25–P75) of at least three independent experiments. Statistical analyses were conducted with GraphPad Prism software version 5.0c (La Jolla, CA, USA). The Mann–Whitney test was used for comparisons between two groups, while the Kruskal–Wallis test was used for comparisons between three or more groups. The Wilcoxon matched-pair signed rank test was used for the comparison of leukocyte subtypes between FF and blood. A *P* value < 0.05 was considered to be significant.

## Results

### Cocultures of granulosa cells and leukocytes enhance G-CSF secretion

**Fig. 2:**
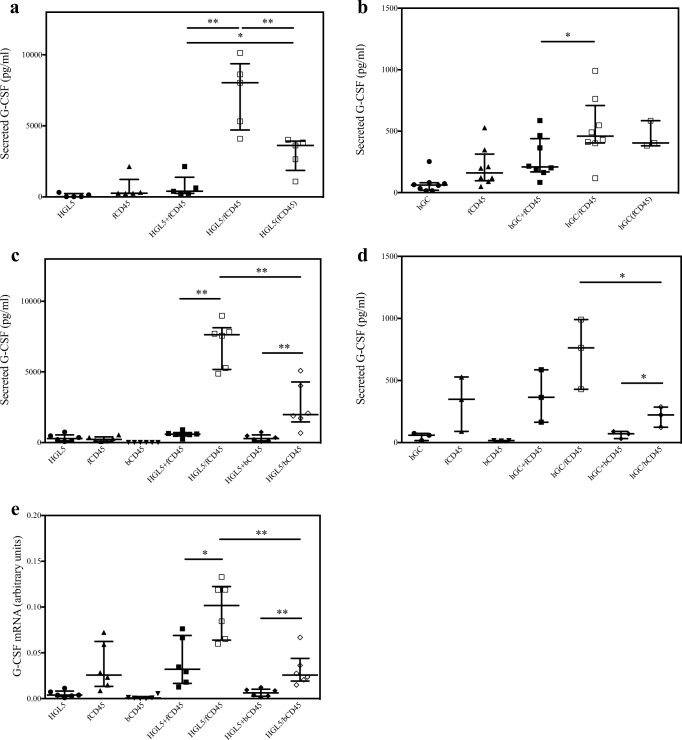
Cocultures of granulosa cells and leukocytes enhance G-CSF secretion. G-CSF secretion (**a**, **b**, **c**, and **d**) and normalized G-CSF mRNA levels (**e**) in 48 h separate cultures, cocultures, and cocultures without cell contact of granulosa cells and leukocytes. HGL5 cells correspond to a human granulosa-derived cell line. hGC and fCD45 cells represent human granulosa cells and leukocytes isolated from FF of women undergoing IVF, while bCD45 cells represent leukocytes obtained from the peripheral blood of the same women. In each experiment, hGC were cocultured with fCD45 or bCD45 cells isolated from the same woman. HGL5 + fCD45, HGL5 + bCD45, hGC + fCD45, and hGC + bCD45 represent the algebraic sum of G-CSF secreted by 5 × 10^5^ HGL5 or hGC cells and 5 × 10^5^ fCD45 or bCD45 cells cultured separately. HGL5/fCD45, HGL5/bCD45, hGC/fCD45, and hGC/bCD45 represent cocultures of 5 × 10^5^ HGL5 or hGC cells with 5 × 10^5^ fCD45 or bCD45 cells. HGL5(fCD45) and hGC(fCD45) represent cocultures of 5 × 10^5^ HGL5 or hGC cells with 5 × 10^5^ fCD45 cells without cell contact, which were performed using plate inserts; granulosa cells were seeded into the bottom chamber and fCD45 cells were seeded into the upper chamber. The results are presented as scatter dot plots. Each symbol represents an independent experiment (*n* = 5 (**a**); *n* = 8 (**b**); *n* = 6 (**c** and **e**) and *n* = 3 (**d**)). The central line indicates the median (50th percentile); the lower and upper bars indicate the 25th and 75th percentiles, respectively. * *P* < 0.05 and ** *P* < 0.01

G-CSF protein, as detected by ELISA assay, was secreted by CD45-negative hGC and fCD45 leukocytes isolated from the FF of women undergoing IVF and by the human granulosa-derived cell line HGL5 after 48 h of culture (Fig. 2a–d). Secreted G-CSF protein was not detected in the 48 h leukocyte cultures (bCD45) of cells from the peripheral blood of 7 out of 8 women undergoing IVF (Fig. 2c and d). The algebraic sum of the amount of G-CSF secreted by 5 × 10^5^ separately cultured HGL5 cells (or hGC) and fCD45 cells (HGL5 + fCD45 or hGC + fCD45) was compared to the amount of G-CSF secreted by the same number of cocultured cells (HGL5/fCD45 or hGC/fCD45). There was a 20-fold increase in the amount of secreted G-CSF when HGL5 cells were cocultured with fCD45 cells (median = 8041 pg/ml for HGL5/fCD45 versus 391.3 pg/ml for HGL5 + fCD45, *P =* 0.008, *n* = 5) (Fig. 2a), and the level of secreted G-CSF doubled when hGC and fCD45 cells were cocultured (median = 460.0 pg/ml for hGC/fCD45 versus 208.7 pg/ml for hGC + fCD45, *P =* 0.04, *n* = 8) (Fig. 2b).

The level of secreted G-CSF was also increased 9-fold when HGL5 cells and fCD45 cells were cocultured in physically separated chambers but with common medium (median = 3622 pg/ml for HGL5(fCD45) versus 391.3 pg/ml for HGL5 + fCD45, *P* = 0.02, *n* = 5), while there was a smaller numerical, but non-statistically significant, increase of 1.9-fold with hGC cells (median = 404.5 pg/ml for hGC(fCD45) versus 208.7 pg/ml for hGC + fCD45, *P* = 0.097, *n* = 3) (Fig. 2a and b).

To assess whether peripheral bCD45 cells have the same ability to enhance G-CSF secretion as fCD45 cells, both bCD45 and fCD45 cells were simultaneously obtained at the time of oocyte retrieval. After 48 h of coculture, G-CSF protein was secreted by isolated HGL5 and fCD45 cells (medians = 284.6 and 221.7 pg/ml, respectively), but not by bCD45 cells. The algebraic sum of G-CSF secreted by 5 × 10^5^ separately cultured HGL5 and fCD45 (or bCD45) cells (HGL5 + fCD45 or HGL5 + bCD45) was compared to the amount of G-CSF secreted by the same number of cocultured cells (HGL5/fCD45 or HGL5/bCD45). G-CSF secretion was significantly increased in both cocultures (median = 7637 pg/ml for HGL5/fCD45 versus 585.5 pg/ml for HGL5 + fCD45, *P* = 0.002 and 1978 pg/ml for HGL5/bCD45 versus 284.6 pg/ml for HGL5 + bCD45, *P* = 0.004, *n* = 6), but the increase in G-CSF secretion was higher in cocultures of HGL5 cells with fCD45 cells than with bCD45 cells (*P* = 0.004) (Fig. 2c). Similar trends were also obtained when G-CSF secretion was evaluated in cocultures of hGC and fCD45 or bCD45 cells isolated from 3 women undergoing IVF (Fig. 2d). The decreased stimulation achieved in cocultures of granulosa cells with peripheral blood leukocytes compared to that achieved in cocultures with follicular leukocytes indicates that follicular leukocytes have a higher capacity to enhance G-CSF production by granulosa cells.

In order to assess whether there was a transcriptional regulation of G-CSF secretion, G-CSF mRNA expression was evaluated in separate cultures of HGL5, fCD45, and bCD45 cells and in cocultures of HGL5 with fCD45 or bC45 cells. G-CSF mRNA was expressed by HGL5 cells, fCD45 cells, and bCD45 cells from 4 out of 6 women, where it represented only 2 to 7% of the G-CSF mRNA isolated from fCD45 cells of the same women. As for G-CSF protein secretion, G-CSF mRNA expression was significantly increased in cocultures of HGL5 and fCD45 or bCD45 cells, and the increase in G-CSF mRNA expression was higher in cocultures of HGL5 with fCD45 cells than with bCD45 cells (Fig. 2e).

### Comparison of leukocyte subtypes in the FF and peripheral blood samples of women undergoing IVF

**Fig. 3:**
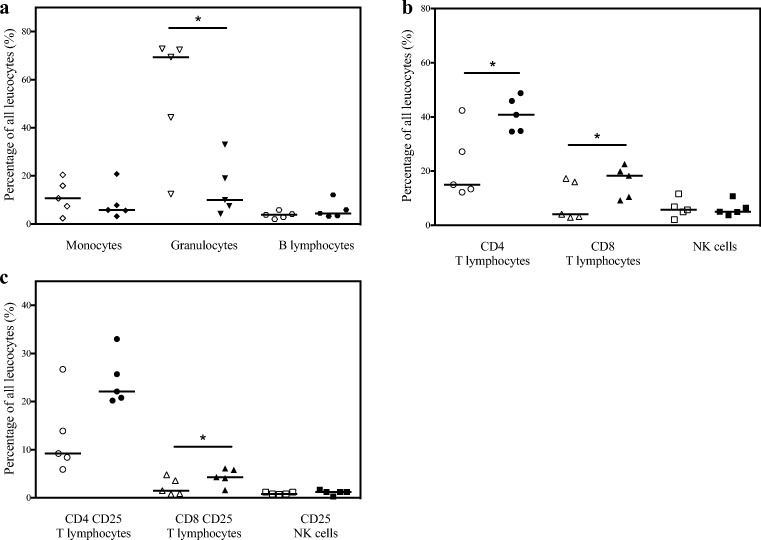
Comparison of leukocyte subtypes in the FF and peripheral blood samples of women undergoing IVF. The percentages of leukocyte subtypes in the total leukocyte population (CD45-positive cells) are shown in follicular fluid (*white symbols*) and in peripheral blood (*black symbols*) samples (*n* = 5). The results are presented as scatter dot plots, with the horizontal line showing the median. (**a**) Monocytes, granulocytes, and B lymphocytes. (**b**) CD4 T lymphocytes, CD8 T lymphocytes, and NK cells. (**c**) CD25-positive CD4 T lymphocytes, CD8 T lymphocytes, and NK cells. * *P* < 0.05

FACS analyses were conducted to compare leukocyte subtypes isolated from FF or the peripheral blood of 5 women at the time of oocyte retrieval. The gating strategy is depicted in Fig. S1 (Supplemental data). The percentage of granulocytes was significantly increased in follicular leukocytes (Fig. 3a, median = 69.4 versus 10.0%, *P* = 0.03), whereas the percentages of CD4 and CD8 T lymphocytes were significantly decreased in the population of follicular leukocytes (Fig. 3b, median = 15.0 versus 40.8%, *P* = 0.03 for CD4 cells and median = 4.1 versus 18.3%, *P* = 0.03 for CD8 cells). The percentage of activated CD25 CD8 T lymphocytes was also decreased in follicular leukocyte samples (Fig. 3c, median = 1.5 versus 4.3%, *P* = 0.03). The proportions of natural killers (NK) cells, B lymphocytes, and monocytes in the populations of blood and follicular leukocytes were similar.

### Granulosa cells are the main source of G-CSF

**Fig. 4:**
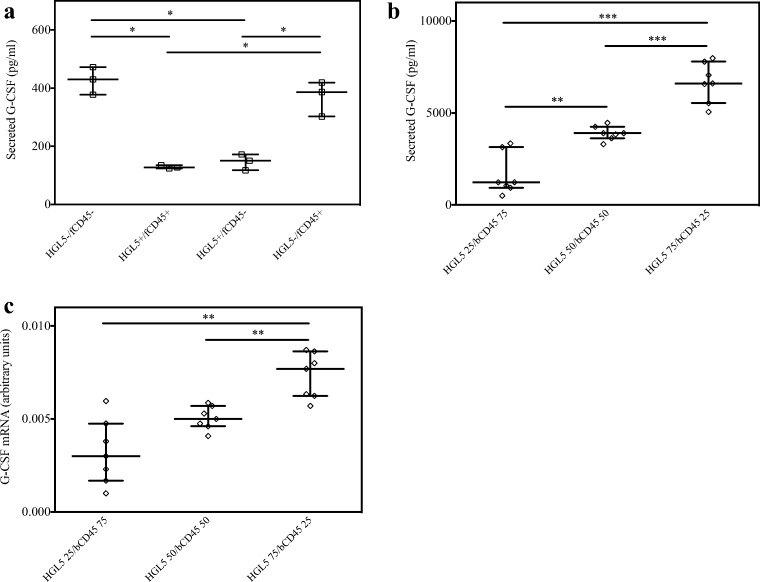
Granulosa cells are the main source of G-CSF. (**a**) G-CSF secretion in cocultures of HGL5 and fCD45 cells after exposure to monensin. HGL5 cells correspond to a human granulosa-derived cell line, and fCD45 cells represent leukocytes isolated from FF of women undergoing IVF. HGL5 and fCD45 cells were individually exposed to monensin overnight. Then, the media were removed and 4 cocultures of HGL5 and fCD45 cells were incubated for 8 h: untreated control coculture with no previous exposure to monensin (HGL5−/fCD45−); coculture with both cell types pretreated with monensin (HGL5+/fCD45+); coculture with monensin-pretreated HGL5 cells and untreated control fCD45 cells (HGL5+/fCD45−); and coculture of untreated control HGL5 cells and monensin-pretreated fCD45 (HGL5−/fCD45+). (**b**) G-CSF secretion and (**c**) normalized G-CSF mRNA levels in cocultures of HGL5 and bCD45 cells isolated from the peripheral blood of women with spontaneous ovulatory cycles. HGL5 and bCD45 cells were cocultured for 24 h with different proportions of each cell type: 2.5 × 10^5^ HGL5 and 7.5 × 10^5^ bCD45 cells (HGL5 25/bCD45 75); 5 × 10^5^ HGL5 and 5 × 10^5^ bCD45 cells (HGL5 50/bCD45 50); 7.5 × 10^5^ HGL5 and 2.5 × 10^5^ bCD45 cells (HGL5 75/bCD45 25). The results are presented as scatter dot plots. Each symbol represents an independent experiment (*n* = 3 (**a**) and *n* = 7 (**b** and **c**)). The central line indicates the median (50th percentile); the lower and upper bars indicate the 25th and 75th percentiles, respectively. ** *P* < 0.01 and *** *P* < 0.001

HGL5 cells and follicular leukocytes (fCD45) were individually treated overnight with monensin to inhibit protein transport and hence, G-CSF secretion in the culture media. After monensin removal, HGL5 and fCD45 cells were washed and cocultured for an additional 8 h. The inhibition of G-CSF secretion after overnight exposure to monensin was confirmed by ELISA (data not shown). This experiment was performed with fCD45 (and not bCD45) cells, since bCD45 cells produce far less G-CSF than fCD45 cells (Fig. 2c and d) and thus do not allow the detection of secreted G-CSF in short coculture experiments of 8 h. When both cell types were pretreated with monensin, G-CSF secretion decreased in the treated cells compared with that in the untreated control cocultured cells (median = 127.4 pg/ml for HGL5+/fCD45+ versus 430.3 pg/ml for HGL5−/fCD45−, *P* = 0.05) (Fig. 4a). In cocultures of monensin-pretreated HGL5 cells and untreated control fCD45 cells, the level of G-CSF secretion was similar to that of cocultures when both cell types were pretreated with monensin (median = 150.3 pg/ml for HGL5+/fCD45− versus 127.4 pg/ml for HGL5+/fCD45+, *P* = 0.35). However, in cocultures of untreated control HGL5 cells and monensin-pretreated fCD45 cells, the G-CSF levels were similar to those of the untreated control cocultures (median = 386.6 pg/ml for HGL5−/fCD45+ versus 430.3 pg/ml for HGL5−/fCD45−, *P* = 0.2).

Since G-CSF secretion is also enhanced in cocultures of HGL5 and bCD45 cells isolated from women at the time of oocyte retrieval, we next performed coculture experiments with bCD45 cells isolated from women with spontaneous ovulatory cycles. Cocultures were performed for 24 h with the following proportions of each cell type: HGL5 cells, 25% and bCD45 cells, 75%; HGL5 cells, 50% and bCD45 cells, 50%; or HGL5 cells 75% and bCD45 cells, 25%. G-CSF secretion and mRNA expression by bCD45 cells were below the detection limits. Both the mRNA and secreted protein levels of G-CSF significantly increased as the percentage of HGL5 cells increased in the coculture (Fig. 4b and c).

We also performed immunohistochemical staining of G-CSF in human ovarian tissue. G-CSF staining was mainly localized to the granulosa cells of secondary, preantral and antral follicles in ovarian cortex fragments. The oocytes were also positive for G-CSF staining (supplemental material and Fig. S2).

Altogether, these results suggest that granulosa cells are the main source of G-CSF secretion and that CD45 cells in cocultures stimulate G-CSF secretion by granulosa cells.

### Effect of the menstrual cycle phase on the expression level of G-CSF

**Fig. 5:**
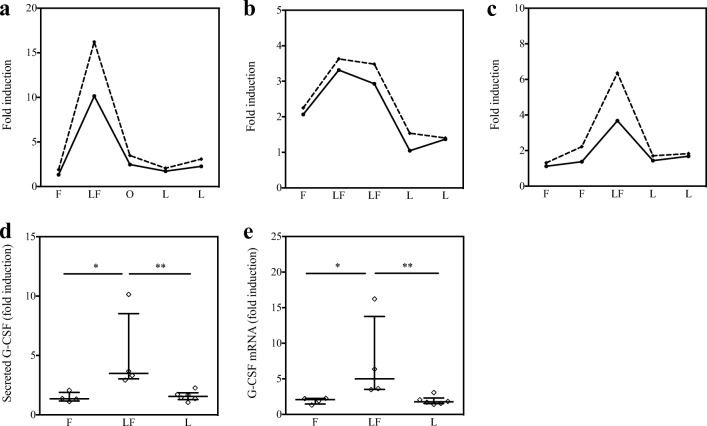
Influence of the menstrual cycle phase on the expression level of G-CSF. G-CSF secreted protein and mRNA were quantified in the 48 h cocultures of HGL5 and bCD45 cells isolated from the peripheral blood of 3 women at different phases of one menstrual cycle. (**a**, **b**, and **c**) Induction of G-CSF mRNA expression (*squares and dotted lines*) and G-CSF secretion (*dots and continuous lines*) for subjects 1, 2, and 3 according to the menstrual cycle phase (F, follicular; LF, late follicular; O, ovulation; L, luteal). The results are expressed as the fold change that represents the ratio between the amount of secreted G-CSF (or G-CSF mRNA) in the coculture of 5 × 10^5^ HGL5 cells and 5 × 10^5^ bCD45 cells (HGL5/bCD45) and the algebraic sum of secreted G-CSF (or G-CSF mRNA) in separate cultures of 5 × 10^5^ HGL5 and 5 × 10^5^ bCD45 cells (HGL5 + bCD45): HGL5/bCD45 divided by (HGL5 + bCD45). Induction of G-CSF secreted protein (**d**) and mRNA (**e**) in cocultures of 5 × 10^5^ HGL5 and 5 × 10^5^ bCD45 cells according to the menstrual cycle phase. The results are presented as scatter dot plots. Each symbol represents an independent experiment (*n* = 14). The central line indicates the median (50th percentile); the lower and upper bars indicate the 25th and 75th percentiles, respectively. * *P* < 0.05 and ** *P* < 0.01

To determine the possible effect of the menstrual cycle phase on G-CSF expression, HGL5 cells were cultured in the presence of peripheral blood leukocytes isolated from 3 women during the follicular, late follicular, ovulatory, or luteal phases of one menstrual cycle. The menstrual cycle phase was determined based on the serum levels of estradiol, LH and progesterone (Table S1). The induction of G-CSF secreted protein and mRNA in cocultures of HGL5 and bCD45 cells is shown throughout one menstrual cycle for each woman (Fig. 5a–c). In the cocultures, the induction of G-CSF secreted protein and mRNA was significantly higher in the late follicular (LF) phase, than in the follicular (F) or luteal (L) phase of the menstrual cycle for the 3 women (G-CSF secreted protein: median = 3.5-fold for LF versus 1.4-fold for F, *P* = 0.03; median = 3.5-fold for LF versus 1.6-fold for L, *P* = 0.01, Fig. 5d and G-CSF mRNA: median = 5.0-fold for LF versus 2.1-fold for F, *P* = 0.03; median = 5.0-fold for LF versus 1.8-fold for L, *P* = 0.01, Fig. 5e).

### The paracrine stimulation of G-CSF secretion by leukocytes is specific

The levels of G-CSF, GROα, IL-6, MCP-1, TNFα, MIP-1α, MIP-1β, MDC, IL-8, and TGF-β1 were measured in the conditioned media of HGL5 and fCD45 cells, cocultures of HGL5 and fCD45 cells or FFDC cells from 7 women undergoing IVF (Table S3). The other 14 cytokines/chemokines tested were not detected in any of the conditioned media.

Only GROα, IL-6, and MCP-1 were secreted by HGL5 cells, fCD45 cells, and FFDC cells, and their levels were upregulated in cocultures of HGL5 and fCD45 cells. These results were confirmed by specific quantitative ELISA for these 3 cytokines/chemokines and G-CSF. Their secretion was increased in cocultures of HGL5 and fCD45 cells compared with separate cultures of HGL5 and fCD45 (*P* = 0.006, *n* = 7) (Fig. S3a). The levels of secreted G-CSF and GROα were significantly higher in FFDC than in hGC and fCD45 cells cultured separately (*P* = 0.03, *n* = 4) (Fig. S3b). The proportions of CD45-negative hGC and CD45-positive fCD45cells in the in FFDC population were 70/30, 54/46, 49/51, and 77/23 for the 4 women undergoing IVF (median = 62/38).

In summary, of the 24 chemokines/cytokines tested, only G-CSF and GROα were modulated by paracrine interactions between follicular leukocytes and human granulosa cells, confirming the specificity of such interactions.

## Discussion

Follicular G-CSF is predictive of oocyte competence and embryo implantation potential in clinical settings [28]. However, its role in oocyte maturation remains poorly understood. Makinoda et al. suggested that follicular G-CSF could induce leukocyte accumulation and activation in the pre-ovulatory follicle and could thus be essential for successful ovulation [29]. In this study, we evaluated the cells and mechanisms regulating G-CSF production in developing follicles.

G-CSF secretion was significantly increased in cocultures of follicular leukocytes with human granulosa cells or with the HGL5 granulosa cell line compared with that in separate cell cultures (Fig. 2a and b). The persistent but lower increase in G-CSF secretion when cocultured cells were physically separated by an insert indicates that direct cell-cell contacts and soluble factors or exosomes could be involved in the paracrine regulation of G-CSF secretion [30, 31]. Polec et al. previously found that the secretion of angiogenic factors was increased in cocultures of granulosa cells with monocytes isolated from the blood of women undergoing IVF [32].

G-CSF secretion was also enhanced when hGC or the HGL5 granulosa cell line were cocultured with blood leukocytes, confirming the specific role of leukocytes in the increase in G-CSF secretion. However, G-CSF production was significantly lower when granulosa cells were cocultured with blood leucocytes than when they were cocultured with follicular leucocytes isolated from the same woman at the time of oocyte retrieval. The considerably higher percentage of granulocytes (70%) isolated from FF than from the peripheral blood (10%) of the same woman at the time of oocyte retrieval (Fig. 3a) might be responsible for the increased stimulation of G-CSF production in cocultures of granulosa cells and follicular leukocytes versus that in cocultures of granulosa cells and peripheral blood leukocytes (Fig. 2c and d). Our results support previous observations that leukocyte populations in both types of biological fluid are distinct [33–35]. Since neutrophils are the most abundant type of granulocyte, our results support the previous finding of the specific accumulation of neutrophils in the preovulatory follicle [22]. In a model of in vitro*-*perfused rat ovaries, the addition of peripheral blood leukocytes increased ovulation [36], and a key role for neutrophils has also been confirmed, with diminished ovulation rates in animals depleted of neutrophils [37, 38].

Inhibition of protein secretion was performed in HGL5 cells and/or leukocytes by means of monensin to identify the cell type responsible for most of the G-CSF secretion. G-CSF secretion was not altered in cocultures of untreated control granulosa cells and monensin-pretreated follicular leukocytes (Fig. 4a); however, in cocultures of monensin-pretreated control granulosa cells and untreated follicular leukocytes, G-CSF production was considerably reduced, indicating that granulosa cells are the main source of FF G-CSF. Data from cocultures with different proportions of HGL5 cells and leukocytes confirm that granulosa cells are the main source of FF G-CSF (Fig. 4b and c) and that paracrine interactions between these cells stimulates its production.

Immunohistochemical staining of G-CSF was positive in granulosa cells (Fig. S2), as demonstrated previously in other reports [21, 39]. In primordial follicles, granulosa cells displayed a faint staining intensity compared with further follicular stages, suggesting that G-CSF production commences during folliculogenesis. The oocytes were also slightly positive for G-CSF staining. To the best of our knowledge, this has not been previously described in humans, since immunohistochemical staining was only performed on granulosa cells isolated from FF or on the follicular walls [21, 39]. However, Cai et al. showed that porcine granulosa cells and oocytes express G-CSF mRNA [40].

G-CSF secretion and mRNA expression were maximal in cocultures of the granulosa cell line HGL5 and blood leukocytes obtained at the late follicular phase, just before ovulation (Fig. 5), further suggesting that circulating leukocytes that move to the preovulatory follicle play a role in the mechanism of ovulation. This finding also supports the results of Yanagi et al., who found a 10-fold increase in the expression of G-CSF mRNA in granulosa cells at the late follicular phase [39].

Despite the identification of multiple chemokines and cytokines in the FF, the synthesis and secretion of only G-CSF and GROα were upregulated by paracrine interactions between follicular leukocytes and human granulosa cells (Fig. S3b). Both G-CSF and GROα are found at higher levels in FF than in serum and are secreted by granulosa cells [19, 41, 42] and leukocytes (G-CSF: monocytes, lymphocytes, natural killer cells, and dendritic cells [10, 11]; GROα: monocytes, lymphocytes, and activated neutrophils [43]). GROα is a neutrophil chemoattractant [44]. G-CSF stimulates granulocyte production, maturation, and function and can increase blood neutrophil counts by releasing precursors from the bone marrow [10–12]. It was demonstrated that FF can exert a chemotactic activity towards leukocytes, especially in conceptual cycles [45, 46]. G-CSF and GROα could thus play a role in the accumulation of neutrophils into the follicular wall before ovulation. These cytokines could be involved in the maturation of follicles leading to a successful ovulation.

The results of this study provide insights into the potential role of G-CSF, a new biomarker of oocyte competence and embryo implantation, in the mechanism of oocyte maturation and ovulation. Follicular G-CSF production depends on the paracrine interaction between granulosa cells and leukocytes before ovulation. Our results support the hypothesis that leukocytes, and namely granulocytes, are recruited in the preovulatory follicle, interact with granulosa cells and regulate G-CSF (and GROα) production at the time of ovulation. Altogether these results suggest that follicular G-CSF production is associated with oocyte maturation, further supporting the interest of follicular G-CSF as a new tool for embryo selection in IVF cycles. Despite experimental evidence that GROα secretion is also modulated by paracrine interactions between granulosa cells and leukocytes, no previous clinical study has identified this chemokine as a marker of oocyte competence and embryo implantation.

Our study has some limitations. This is a laboratory research study using an in vitro culture system to test the mechanisms of follicular G-CSF production on a relatively small number of women undergoing IVF (*n* = 41). The increase in G-CSF secretion in the coculture of primary human granulosa cells and leukocytes was reproduced with a granulosa-derived cell line. This cell line was used in further experiments assessing the transcriptional regulation of G-CSF secretion at the mRNA level, the cellular source of G-CSF secretion, and the impact of the menstrual cycle phase. Due to the small sample size, we could not investigate potential confounding factors, such as the cause of infertility or the stimulation protocol on G-CSF secretion. However, we obtained similar results with blood leukocytes isolated from women with spontaneous ovulatory cycles. For ethical reasons, we could not collect FF from fertile women with spontaneous ovulatory cycles. Thus, both the quantification of FF G-CSF and the paracrine interactions between follicular leukocytes and human granulosa cells (or the granulosa cell line HGL5) could not be assessed at various stages of the menstrual cycle. Another limitation is that we have not elucidated which leukocyte subtype is responsible for the induction of G-CSF secretion in cocultures with granulosa cells. We showed that granulocytes are more abundant in the population of follicular versus blood leukocytes, but it remains to be elucidated whether granulocytes are responsible for the induction of follicular G-CSF secretion by granulosa cells.

## Conclusions

In clinical settings, increased follicular G-CSF concentrations are associated with an increased probability of embryo implantation and successful pregnancy. The notable rise in G-CSF at the time of ovulation coincides with the accumulation of follicular granulocytes, most likely neutrophils, which stimulate G-CSF production by granulosa cells via paracrine interactions. This mechanism seems to be specific since it is not observed with a range of other FF cytokines and chemokines, except for GROα.

## Electronic supplementary material

ESM 1 Figure S1 FACS gating strategy. Cells were isolated from the FF and the peripheral blood of women undergoing IVF on the day of oocyte pick-upFigure S2 Immunohistochemical staining of G-CSF in human ovarian tissue. Scale bar: 250 μm (**a**). The plain arrow indicates a primordial follicle, which consists of the oocyte surrounded by a single layer of squamous (pre)granulosa cells (magnification (**b**) and negative control (**e**)). The arrowhead shows a secondary follicle, that is recognized by two layers of cuboidal granulosa cells surrounding the oocyte (magnification (**c**) and negative control (**f**)). The triangle indicates a preantral follicle with multiple layers of granulosa cells (magnification (**d**) and negative control (**g**)). Magnification scale bar: 50 μm. Figure S3 Secretion of G-CSF, GROα, IL-6, and MCP-1 in cultures of HGL5 cells, fCD45 cells, hGC, and FFDC. (**a**) Secretion of G-CSF, GROα, IL-6, and MCP-1 in separately cultured and in cocultured HGL5 and fCD45 cells. (**b**) Secretion of G-CSF, GROα, IL-6, and MCP-1 in separately cultured hGC and fCD45 cells and in FFDC cells. HGL5 cells correspond to a human granulosa-derived cell line. FFDC cells were obtained after enzymatic digestion of the FF followed by a density gradient centrifugation over a Ficoll-Paque Plus gradient. hGC and fCD45 cells were further isolated after magnetic-activated cell sorting of FFDC and represent human granulosa cells and follicular leukocytes respectively. FFDC cells were made of 70/30, 54/46, 49/51, and 77/23% of hGC and fCD45 cells in the 4 independent experiments, after FACS analysis (**b**). The algebraic sum of the secreted levels of cytokines/chemokines in separate 48-h cultures of 5 × 10^5^ HGL5 (or hGC) and 5 × 10^5^ fCD45 (HGL5 + fCD45 or hGC + fCD45) are represented by black symbols. White symbols represent the secretion of these cytokines/chemokines in the 48 h coculture of 5 × 10^5^ HGL5 and 5 × 10^5^ fCD45 cells (HGL5/fCD45) (**a**) or in the 48 h culture of 10^6^ FFDC cells (**b**). The results are presented as scatter dot plots. Each symbol represents an independent experiment (*n* = 7 (**a**) and *n* = 4 (**b**)). The central line indicates the median (50th percentile); the lower and upper bars indicate the 25th and 75th percentiles, respectively. The absolute concentrations of IL-6 were divided by 10 in Fig. S3a. * *P* < 0.05 and *** *P* < 0.001 (PDF 2130 kb)

ESM 2 (DOCX 13 kb)

Table S1 (DOCX 20 kb)

Table S2 (DOCX 13 kb)

Table S3 (DOCX 17 kb)
