# Supplementary material for: A paracrine interaction between granulosa cells and leukocytes in the preovulatory follicle causes the increase in follicular G-CSF levels
Source: J Assist Reprod Genet. 2020 Jan 18;37(2):405–16. doi: 10.1007/s10815-020-01692-y (PMC7056696; doi:10.1007/s10815-020-01692-y)
Supplement: Supplementary file 1 — Figure S1 FACS gating strategy. Cells were isolated from the FF and the peripheral blood of women undergoing IVF on the day of oocyte pick-upFigure S2 Immunohistochemical staining of G-CSF in human ovarian tissue. Scale bar: 250 μm (a). The plain arrow indicates a primordial follicle, which consists of the oocyte surrounded by a single layer of squamous (pre)granulosa cells (magnification (b) and negative control (e)). The arrowhead shows a secondary follicle, that is recognized by two layers of cuboidal granulosa cells surrounding the oocyte (magnification (c) and negative control (f)). The triangle indicates a preantral follicle with multiple layers of granulosa cells (magnification (d) and negative control (g)). Magnification scale bar: 50 μm. Figure S3 Secretion of G-CSF, GROα, IL-6, and MCP-1 in cultures of HGL5 cells, fCD45 cells, hGC, and FFDC. (a) Secretion of G-CSF, GROα, IL-6, and MCP-1 in separately cultured and in cocultured HGL5 and fCD45 cells. (b) Secretion of G-CSF, GROα, IL-6, and MCP-1 in separately cultured hGC and fCD45 cells and in FFDC cells. HGL5 cells correspond to a human granulosa-derived cell line. FFDC cells were obtained after enzymatic digestion of the FF followed by a density gradient centrifugation over a Ficoll-Paque Plus gradient. hGC and fCD45 cells were further isolated after magnetic-activated cell sorting of FFDC and represent human granulosa cells and follicular leukocytes respectively. FFDC cells were made of 70/30, 54/46, 49/51, and 77/23% of hGC and fCD45 cells in the 4 independent experiments, after FACS analysis (b). The algebraic sum of the secreted levels of cytokines/chemokines in separate 48-h cultures of 5 × 10^5^ HGL5 (or hGC) and 5 × 10^5^ fCD45 (HGL5 + fCD45 or hGC + fCD45) are represented by black symbols. White symbols represent the secretion of these cytokines/chemokines in the 48 h coculture of 5 × 10^5^ HGL5 and 5 × 10^5^ fCD45 cells (HGL5/fCD45) (a) or in the 48 h culture of 106 FFDC cells (b). The results are presen [file 10815_2020_1692_MOESM1_ESM.pdf]

Figure S1 (1)

Panel A

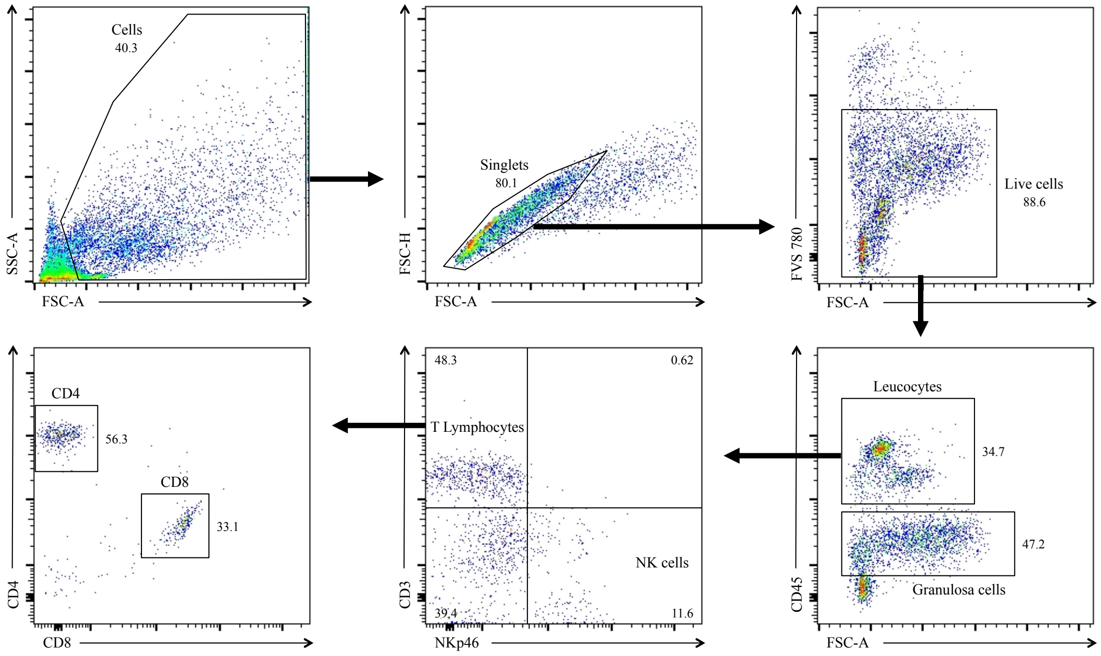

Panel B
Gate on leukocytes (live singlets, CD45 cells)

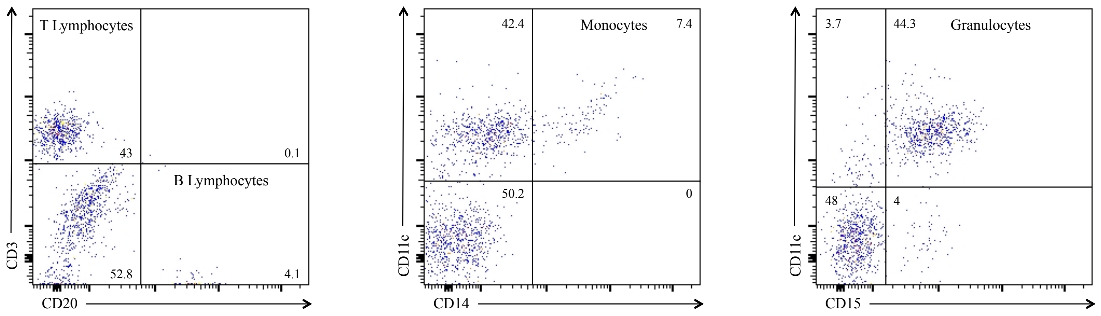

Figure S1 (2)

Panel A : identification of activated CD25 cells

Gate on CD4 T lymphocytes (live singlets, CD45 CD3 CD4 cells)

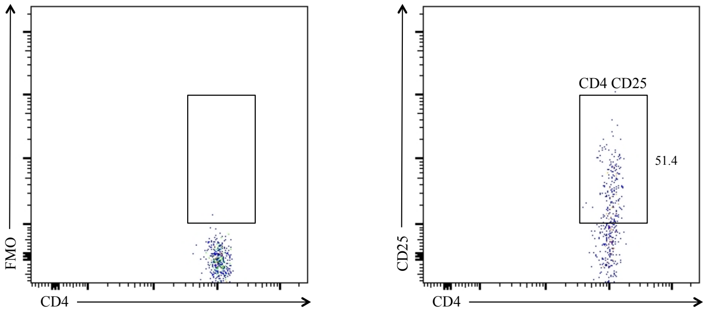

Gate on CD8 T lymphocytes (live singlets, CD45 CD3 CD8 cells)

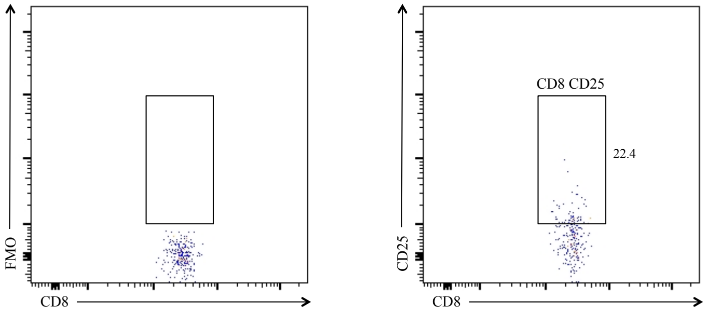

Gate on NK cells (live singlets, CD45 NKp46 cells)

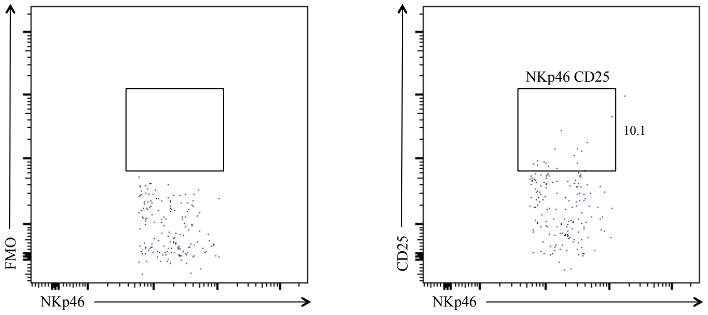

Figure S2

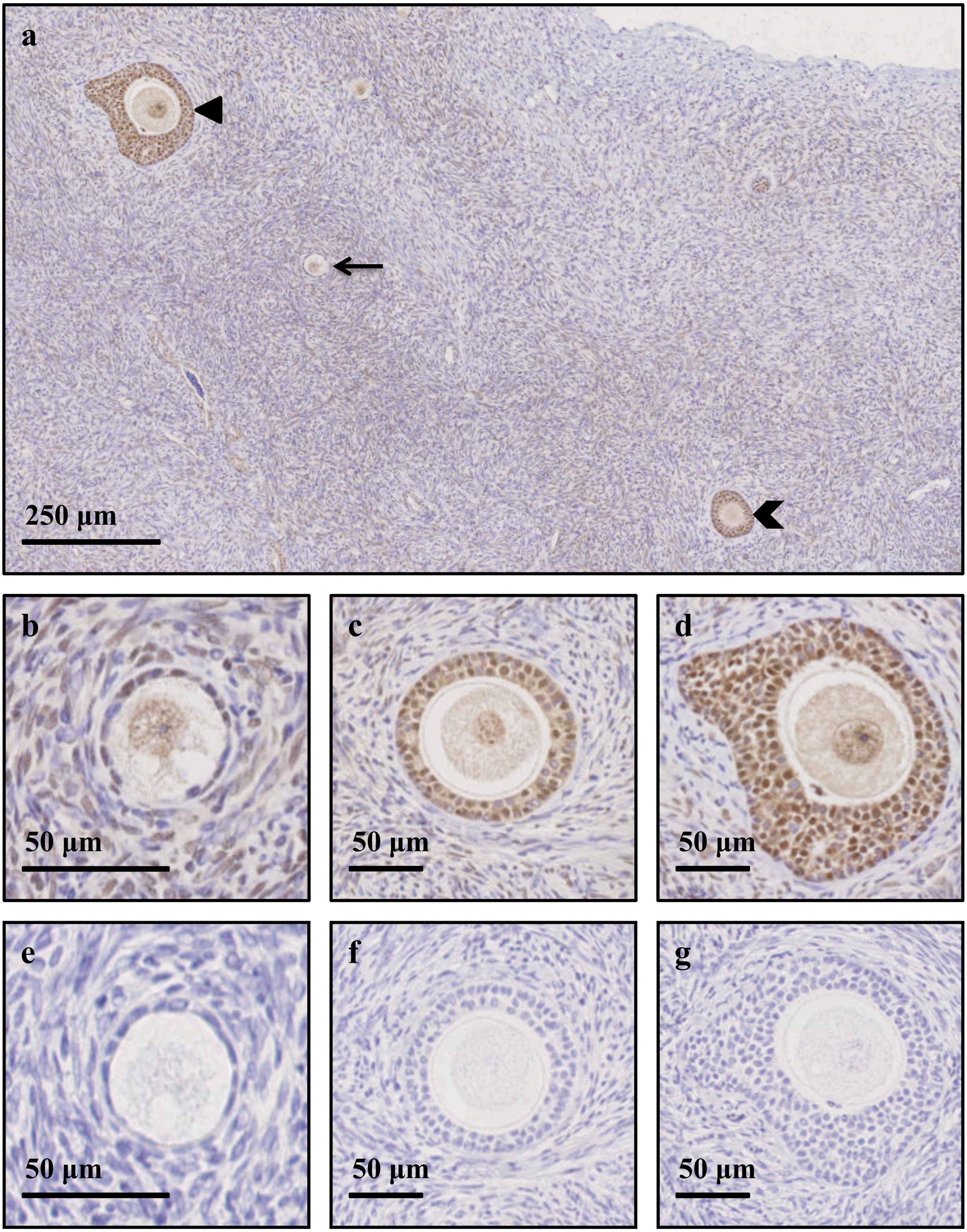

Figure S3

a

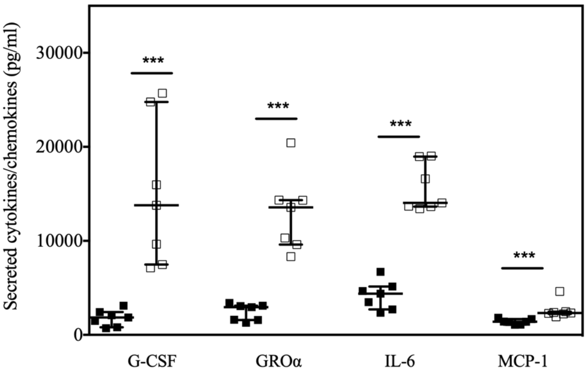
Secreted cytokines/chemokines (pg/ml)
30000
20000
10000
0
***
***
***
***
G-CSF
GROα
IL-6
MCP-1

b

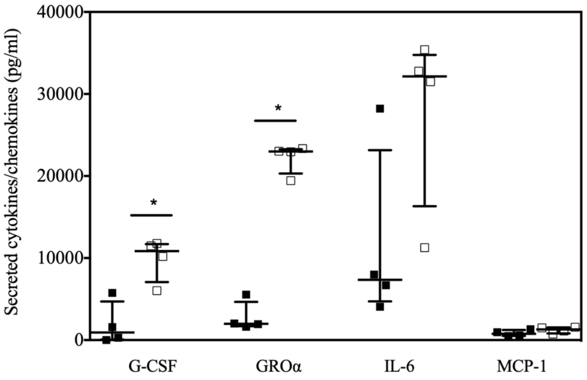
Secreted cytokines/chemokines (pg/ml)
40000
30000
20000
10000
0
*
*
G-CSF
GROα
IL-6
MCP-1
