# Supplementary material for: A paracrine interaction between granulosa cells and leukocytes in the preovulatory follicle causes the increase in follicular G-CSF levels
Source: J Assist Reprod Genet. 2020 Jan 18;37(2):405–16. doi: 10.1007/s10815-020-01692-y (PMC7056696; doi:10.1007/s10815-020-01692-y)
Supplement: Supplementary file 2 — (DOCX 13 kb) [file 10815_2020_1692_MOESM2_ESM.docx]

**Supplemental Material**

**Immunohistochemical staining of G-CSF in human ovarian tissue (Fig. S2)**

Human ovarian tissue was obtained from 2 reproductive age organ donors (30 and 38 years

old). The use of ovarian tissue was approved by the Ethics Committee of the CHU, Liege University (CE707/2014-282). Ovarian fragments were fixed in 4% formaldehyde, embedded in paraffin and cut into 5 μm-thick serial sections. Antigen retrieval was performed with citrate 1:10 (Dako,Glostrup, Denmark, S2031) and unspecific binding sites were blocked with protein block serum-free ready-to-use (Dako, X0909). G-CSF was immunolabeled with the rabbit polyclonal anti-human G-CSF antibody (Abcam, Cambridge, UK, Ab9691) at a dilution of 1:1000 overnight at 4°C. We used the EnVision + System-HRP labeled polymer anti-rabbit ready-to-use (Dako, K4003) as the secondary antibody for 30 min at room temperature. The 3,3′-diaminobenzidine substrate (Dako, K3468) allowed the visualization of the staining and the sections were counterstained with hematoxylin. Negative control slides were obtained by replacing the primary antibody with phosphate-buffered saline (PBS), while testicles slides were used as positive controls. Slides were digitized as previously described (Fransolet et al. 2015).

**Secretion of G-CSF, GROα, IL-6 and MCP-1 in cultures of HGL5, fCD45, hGC and FFDC cells (Table S3 and Fig. S3)**

In order to assess the secretion of various cytokines and chemokines in cocultures of granulosa cells and follicular leukocytes, FFDC, hGC and fCD45 cells were isolated from FF of women undergoing IVF. For separate cultures, 5x10^5^ HGL5 or hGC or fCD45 cells were seeded in 12-well plates at a density of 5x10^5^ cells per well. For cocultures, 5x10^5^ HGL5 were seeded with 5x10^5^ fCD45 cells per well. Cultures of 10^6^ FFDC cells were also performed. After 48 h, conditioned media were collected: the Human Th1/Th2/Th17 cytokines and the Human common chemokines multi-analyte ELISArray kits were used for the qualitative evaluation of various secreted human cytokines and chemokines (Table S3) and specific ELISA assays were used for the quantification of secreted G-CSF, GROα, IL-6 and MCP-1 (Fig. S3).
