# Supplementary material for: A paracrine interaction between granulosa cells and leukocytes in the preovulatory follicle causes the increase in follicular G-CSF levels
Source: J Assist Reprod Genet. 2020 Jan 18;37(2):405–16. doi: 10.1007/s10815-020-01692-y (PMC7056696; doi:10.1007/s10815-020-01692-y)
Supplement: Supplementary file 3 — (DOCX 20 kb) [file 10815_2020_1692_MOESM3_ESM.docx]

**Table S1** Hormonal levels throughout the menstrual cycle

| **Menstrual cycle** | | **Estradiol**  (pg/ml) | **Luteinizing**  **hormone** (U/l) | **Progesterone**  (μg/l) |
| --- | --- | --- | --- | --- |
| **Day** | **Phase** |  |  |  |
| **Women 1** |  |  |  |  |
| 14 | follicular | 49 | 6 | <0.17 |
| 17 | late follicular | 100 | 9.9 | <0 .17 |
| 21 | ovulation | 490 | 55 | 0.53 |
| 24 | luteal | 106 | 11.1 | 3.49 |
| 31 | luteal | 105 | 1.3 | 6.2 |
| **Women 2** |  |  |  |  |
| 3  10 | follicular | 50 | 3.5 | 0.81 |
| 10 | late follicular | 106 | 7 | <0.17 |
| 14 | late follicular | 316 | 10.1 | <0.17 |
| 17 | luteal | 127 | 11.5 | 2.11 |
| 24 | luteal | 160 | 1.4 | 13.08 |
| **Women 3** |  |  |  |  |
| 4 | follicular | 26 | 6.8 | <0.17 |
| 11 | follicular | 86 | 8.8 | <0.17 |
| 14 | late follicular | 281 | 7.6 | <0.17 |
| 18 | luteal | 138 | 6.2 | 5.81 |
| 21 | luteal | 268 | 5.2 | 17.46 |
| **Laboratory references** | |  |  |  |
| Follicular | | 26-99 | 1.0-20 | 0.2-1.5 |
| Late follicular | | 100-156 | 1.0-20 | 0.2-1.5 |
| Ovulation | | 48-314 | 24-105 | 0.8-3 |
| Luteal | | 33-300 | 0.5-20 | 1.7-27 |
