# Supplementary material for: A paracrine interaction between granulosa cells and leukocytes in the preovulatory follicle causes the increase in follicular G-CSF levels
Source: J Assist Reprod Genet. 2020 Jan 18;37(2):405–16. doi: 10.1007/s10815-020-01692-y (PMC7056696; doi:10.1007/s10815-020-01692-y)
Supplement: Supplementary file 4 — (DOCX 13 kb) [file 10815_2020_1692_MOESM4_ESM.docx]

| **Immunogen** | **Fluorochrome** | **Clone** | **Dilution** | **Panel** |
| --- | --- | --- | --- | --- |
| CD45 | FITC | HI30 | 1:5 | A/B |
| CD3 | BV510 | HIT3A | 1:20 | A/B |
| CD4 | PE-Cy 7 | SK3 | 1:20 | A |
| CD8 | PerCP-Cy 5.5 | SK1 | 1:20 | A |
| CD25 | BV421 | M-A251 | 1:20 | A |
| CD335 (NKp46) | APC | 9E2/Nkp46 | 1:5 | A |
| CD11c | BV421 | B-ly6 | 1:20 | B |
| CD14 | APC | M5E2 | 1:5 | B |
| CD20 NHP | PerCP-Cy 5.5 | 2H7 | 1:20 | B |
| CD15 | PE-Cy7 | HI98 | 1:20 | B |

**Table S2** Human antibodies used for flow cytometry (BD Biosciences)
