# Supplementary material for: A paracrine interaction between granulosa cells and leukocytes in the preovulatory follicle causes the increase in follicular G-CSF levels
Source: J Assist Reprod Genet. 2020 Jan 18;37(2):405–16. doi: 10.1007/s10815-020-01692-y (PMC7056696; doi:10.1007/s10815-020-01692-y)
Supplement: Supplementary file 5 — (DOCX 17 kb) [file 10815_2020_1692_MOESM5_ESM.docx]

|  | **Optical densities** | | | |
| --- | --- | --- | --- | --- |
|  | **HGL5** | **fCD45** | **HGL5/fCD45** | **FFDC** |
| **G-CSF** | 0 | 0.001 | 0.64 | 1.28 |
| **GROα** | 0.08 | 0.13 | 2.71 | 3.78 |
| **IL-6** | 4.14 | 0.34 | 4.74 | 2.23 |
| **MCP-1** | 0.50 | 0.05 | 2.51 | 0.20 |
| **TNFα** | 0 | 0.47 | 0.38 | 0.37 |
| **MIP-1α** | 0 | 0.41 | 0.07 | 0.61 |
| **MIP-1β** | 0 | 0.35 | 0 | 0.63 |
| **MDC** | 0 | 0.28 | 0.08 | 0.54 |
| **IL-8** | 1.51 | 1.76 | 1.64 | 1.87 |
| **TGF-β1** | 0.93 | 0.07 | 0.68 | 0.22 |

**Table S3** Detectable cytokines/chemokines in cultures of HGL5 and fCD45 cells, in cocultures of HGL5 and fCD45 cells (HGL5/fCD45) and in follicular fluid-derived cells (FFDC) analysed with the Human Th1/Th2/Th17 cytokines and the Human common chemokines multi-analyte ELISArray kits
